# Sustained activation of the FGF1–MEK–ERK pathway inhibits proliferation, invasion and migration and enhances radiosensitivity in mouse angiosarcoma cells

**DOI:** 10.1093/jrr/rrae021

**Published:** 2024-04-18

**Authors:** Taichi Miura, Junko Kado, Kazuma Ashisuke, Mikio Masuzawa, Fumiaki Nakayama

**Affiliations:** Regenerative Therapy Research Group, Department of Radiation Regulatory Science Research, National Institute of Radiological Sciences (NIRS), National Institutes for Quantum Science and Technology (QST), 4-9-1 Anagawa, Inage-ku, Chiba 263-8555, Japan; Regenerative Therapy Research Group, Department of Radiation Regulatory Science Research, National Institute of Radiological Sciences (NIRS), National Institutes for Quantum Science and Technology (QST), 4-9-1 Anagawa, Inage-ku, Chiba 263-8555, Japan; Radiation Effect Research Group, Department of Accelerator and Medical Physics, Institute for Quantum Medical Science, National Institutes for Quantum Science and Technology (QST), 4-9-1 Anagawa, Inage-ku, Chiba 263-8555, Japan; Department of Dermatology, Iwase General Hospital, 20 Kitamachi, Sukagawa-shi, Fukushima 962-8503, Japan; Regenerative Therapy Research Group, Department of Radiation Regulatory Science Research, National Institute of Radiological Sciences (NIRS), National Institutes for Quantum Science and Technology (QST), 4-9-1 Anagawa, Inage-ku, Chiba 263-8555, Japan

**Keywords:** FGF1, angiosarcoma cells, actin polymerization, MEK inhibitor, radiosensitivity

## Abstract

Angiosarcoma is a rare refractory soft-tissue tumor with a poor prognosis and is treated by radiotherapy. The fibroblast growth factor 1 (FGF1) mutant, with enhanced thermostability due to several substituted amino acids, inhibits angiosarcoma cell metastasis, yet the mechanism of action is unclear. This study aims to clarify the FGF1 mutant mechanism of action using ISOS-1 mouse angiosarcoma cells. The wild-type FGF1 or FGF1 mutant was added to ISOS-1 cells and cultured, evaluating cell numbers over time. The invasive and migratory capacity of ISOS-1 cells was assessed by transwell analysis. ISOS-1 cell radiosensitivity was assessed by colony formation assay after X-ray irradiation. To examine whether mitogen-activated protein kinase (MEK) inhibitor counteracts the FGF1 mutant effects, a combination of MEK inhibitor and FGF1 mutant was added to ISOS-1 cells and cultured. The FGF1 mutant was observed to inhibit ISOS-1 cell proliferation, invasion and migration by sustained FGF1 signaling activation. A MEK inhibitor suppressed the FGF1 mutant-induced inhibition of proliferation, invasion and migration of ISOS-1 cells. Furthermore, the FGF1 mutant enhanced radiosensitivity of ISOS-1 cells, but MEK inhibition suppressed the increased radiosensitivity. In addition, we found that the FGF1 mutant strongly inhibits actin polymerization, suggesting that actin cytoskeletal dynamics are closely related to ISOS-1 cell radiosensitivity. Overall, this study demonstrated that in ISOS-1 cells, the FGF1 mutant inhibits proliferation, invasion and migration while enhancing radiosensitivity through sustained activation of the MEK-mediated signaling pathway.

## INTRODUCTION

Angiosarcoma is an extremely rare soft-tissue tumor accounting for ~4–5% of soft-tissue sarcomas [1–3]. The prognosis for angiosarcoma is poor, and the incidence of angiosarcoma is increasing in recent years [3, 4]. Angiosarcomas occur anywhere in the body, including the liver, breast, heart and bones, but most commonly occur on the skin, accounting for half of all angiosarcomas [3, 5]. Relatively early after onset, angiosarcomas metastasize to various organs either hematogenously or via lymphatics, with a very poor prognosis when angiosarcomas metastasize distally [3, 5, 6]. Radiotherapy is an effective angiosarcoma treatment and is administered in combination with surgery or chemotherapy [7–9]. However, angiosarcoma radiation therapy involves high-dose (50–70 Gy) extended-field irradiation, increasing risks of side effects [7–9]. In addition, radiation therapy for angiosarcoma is generally performed after extensive surgical resection, placing an additional heavy burden on the recovering patient. Therefore, the development of angiosarcoma treatments that do not impair patient quality of life (QOL) is required.

The human fibroblast growth factor (FGF) family contains 22 members, some of which have protective effects against radiation-induced damage [10, 11]. Fibroblast growth factor 1 (FGF1) is expected to be clinically used as a radioprotector as it provides greater protection against radiation-induced intestinal damage than other radioprotective FGFs [11]. However, as FGF1 has low thermal stability and a relatively short *in vivo* half-life, there are efforts to improve the FGF1 thermal stability [12–15]. Previous reports demonstrated that mutant FGF1 (FGF1-PIGN), in which four amino acids, Gln-40, Ser-47, His-93 and Lys-112, are replaced by Pro (P), Ile (I), Gly (G) and Asn (N), respectively, has very high thermal stability and provides a significantly greater protective effect against radiation-induced intestinal damage compared with wild-type FGF1 [15]. Also, FGF1-PIGN is reported to enhance the radiosensitivity of mouse angiosarcoma cells, ISOS-1 cells [15]. Furthermore, FGF1-PIGN is also reported to inhibit both the invasive and migratory potential of ISOS-1 cells [15]. Therefore, FGF1-PIGN is potentially a novel agent that promotes radiosensitivity and inhibits angiosarcoma invasion and metastasis while protecting normal tissues, but the FGF1-PIGN mechanism of action is unknown.

This study focused on the cytoplasmic components of FGF1 signaling to clarify the mechanism of action of FGF1-PIGN in ISOS-1 cells. Our results demonstrated that in ISOS-1 cells, FGF1-PIGN inhibits the proliferation, invasion, and migration and enhances radiosensitivity through sustained activation of the MEK-mediated signaling pathway. This study facilitates the development of new angiosarcoma treatments that do not impair patient’s QOL.

## MATERIALS AND METHODS

### Recombinant proteins

**Fig. 1:**
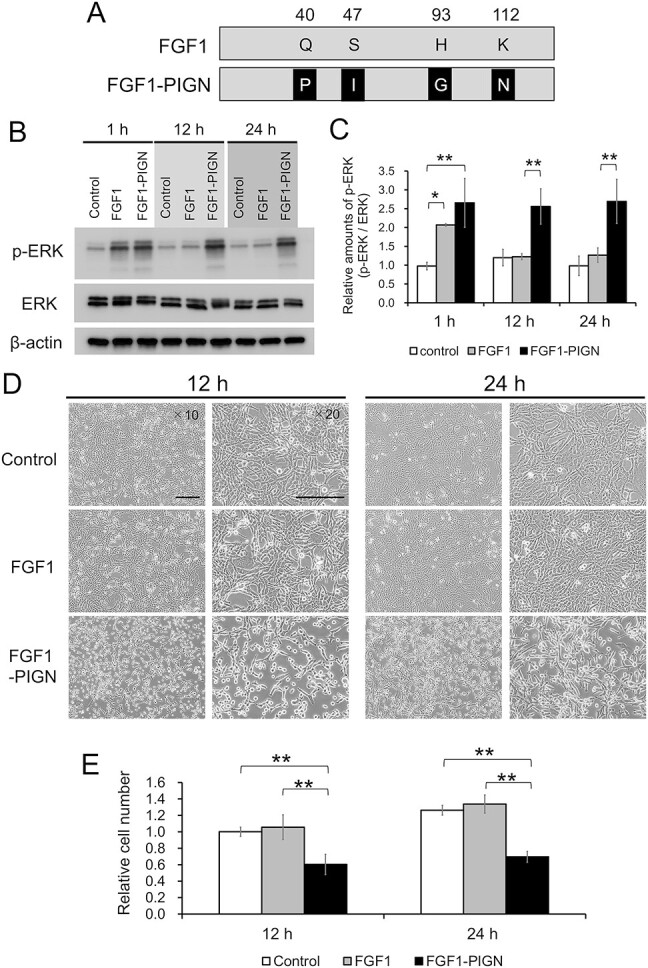
FGF1-PIGN suppresses ISOS-1 cell proliferation through sustained activation of FGF1 signaling. (A) FGF1-PIGN was created by substituting Gln-40, Ser-47, His-93 and Lys-112 for Pro, Ile, Gly and Asn, respectively, in wild-type FGF1. (B) Western blot analysis using antibodies against phosphorylated ERK (p-ERK), ERK and β-actin in ISOS-1 cells cultured for 1, 12 and 24 h with 100 ng/ml wild-type FGF1 or 100 ng/ml FGF1-PIGN. (C) Histograms show the mean densitometric reading ± standard deviation (SD) of p-ERK/ERK after normalization against levels in control cells after cultivation for 1 h (set to 1). (D) The morphology of ISOS-1 cells cultured for 12 and 24 h with 100 ng/ml wild-type FGF1 or 100 ng/ml FGF1-PIGN. Scale bars: 200 μm. (E) Histograms show the mean number of cells ± SD after normalization against the value of control cells at 12 h (set to 1). Representative images of western blot and cell morphology are shown. Values were obtained from three independent experiments. ^*^P < 0.05, and ^**^P < 0.01.

Wild-type human FGF1 and mutant FGF1 (FGF1-PIGN), shown in Fig. 1A, were purified as described previously [15, 16]. Briefly, mutations of the human FGF1 gene were introduced using the QuikChange site-directed mutagenesis kit (Agilent Technologies, Santa Clara, CA). The wild-type FGF1 or FGF1-PIGN gene was transferred into the pDEST17 vector (Thermo Fisher Scientific, Waltham, MA), an N-terminal fusion vector that contains a sequence encoding a 6 × His tag, and the pDEST17 expression constructs were transformed into BL21(DE3)pLysS *Escherichia coli* cells. Protein expression induction was performed using the Overnight Express Autoinduction System 1 (Merck kGaA, Darmstadt, Germany), according to the manufacturer’s instructions. The cell pellets were lysed in BugBuster Master Mix (Merck kGaA) including ethylenediaminetetraacetic acid (EDTA)-free protease inhibitor cocktail (cOmplete ULTRA) (Roche Diagnostics, Mannheim, Germany), and soluble extracts purified using a Ni Sepharose High Performance column (GE Healthcare, Waukesha, WI).

### Inhibitors

PD0325901 (Chemscence, Monmouth Junction, NJ), a MEK inhibitor; Gö 6983 (Abcam, Cambridge, UK), a protein kinase c (PKC) inhibitor; MK-2206 (Selleck, Houston, TX), a protein kinase b (AKT) inhibitor; Losmapimod (Selleck), a p38α/β mitogen-activated protein kinase (p38 MAPK) inhibitor; SP600125 (Selleck), a c-jun N-terminal kinase (JNK) inhibitor; Saracatinib (Selleck), a proto-oncogene tyrosine-protein kinase Src (SRC) inhibitor and JAK Inhibitor I (Santa Cruz Biotechnology, Dallas, TX), a janus kinase (JAK) inhibitor, were dissolved in dimethyl sulfoxide (DMSO) (Sigma, ST. Louis, MO) and added to the culture medium. Pure DMSO was used as the negative control.

### Cell culture

The murine angiosarcoma cell line ISOS-1 [17] was maintained in Dulbecco’s modified Eagle’s medium (DMEM) (Thermo Fisher Scientific) supplemented with 10% heat-inactivated fetal bovine serum (FBS) (Thermo Fisher Scientific).

To examine the effect of wild-type FGF1 or FGF1-PIGN on ISOS-1 cells, 1 × 10^5^ ISOS-1 cells were seeded in 35-mm culture dishes, cultured for 48 h after seeding and then 100 ng/ml wild-type FGF1 or 100 ng/ml FGF1-PIGN was added alone or with 1 μM PD0325901, 1 μM Gö 6983, 5 μM MK-2206, 1 μM Losmapimod, 10 μM SP600125, 1 μM Saracatinib or 0.5 μM JAK Inhibitor I to ISOS-1 cells. Then, ISOS-1 cell morphology was observed over time, recording cell numbers.

### Western blotting

Phosphorylated extracellular signal-regulated kinase (phospho-ERK) levels in ISOS-1 cells incubated with 100 ng/ml wild-type FGF1 or 100 ng/ml FGF1-PIGN alone or with 1 μM PD0325901 for 1, 12 and 24 h were examined by western blot analysis. Cells were washed with phosphate-buffered saline (PBS) then lysed with lysis buffer (50 mM Tris–HCl [pH 7.4], 150 mM NaCl, 1% Triton X-100, phosphatase inhibitor [PhosSTOP] [Roche Diagnostics] and protease inhibitors [cOmplete ULTRA] [Roche Diagnostics]). The lysates were collected using a cell scraper and protein concentration evaluated using the Bradford dye-binding method (Bio-Rad Protein Assay) (Bio-Rad Laboratories, Hercules, CA). Ten micrograms of protein lysate from each sample were separated on a Mini-PROTEAN TGX gel (Bio-Rad Laboratories) and transferred to polyvinylidene fluoride membranes (Millipore, Burlington, MA). After blocking with bovine serum albumin (BSA) (Thermo Fisher Scientific), membranes were incubated with primary antibodies against ERK (Cell Signaling Technology, Danvers, MA), phospho-ERK (Cell Signaling Technology) and β-actin (Cell Signaling Technology) at 4°C overnight. Then, membranes were incubated with HRP-linked anti-rabbit IgG (Cell Signaling Technology) or horseradish peroxidase (HRP)-linked anti-mouse IgG (Cell Signaling Technology), washed and developed with ECL Select reagents (GE Healthcare).

### Immunostaining

Cells were fixed with 4% paraformaldehyde, washed and blocked with PBS containing 20% Block Ace (KAC Co., Tokyo, Japan), 0.3% Triton X-100 and 5% BSA. To detect filamentous actin (F-actin), cells were stained with Alexa Fluor 488-conjugated Phalloidin (Thermo Fisher Scientific) for 2 h at room temperature. Nuclei were stained using Hoechst dye (Thermo Fisher Scientific). After washing with PBS, cells were mounted and visualized using an IX70 fluorescence microscope (Olympus, Tokyo, Japan) equipped with an ORCA-R2 cooled CCD camera (Hamamatsu Photonics, Shizuoka, Japan).

### Invasion and migration assays

Invasion and migration assays using Transwell chambers (Corning, Horseheads, NY) were performed as previously described [15]. Briefly, pre-Matrigel (BD Biosciences, Bedford, MA)-coated culture inserts were used for the invasion assay, and those not coated with Matrigel were used for the migration assay. 5 × 10^4^ ISOS-1 cells per well were seeded using DMEM containing 100 ng/ml wild-type FGF1 or 100 ng/ml FGF1-PIGN alone or with 1 μM PD0325901. After 24-h incubation, cells were stained with Diff Quick (Sysmex, Hyogo, Japan), and the number of invaded or migrated cells was evaluated under a microscope.

### Colony formation assay

Colony formation assays of ISOS-1 cells after X-ray irradiation were performed as previously described [15]. ISOS-1 cells were irradiated with X-rays at doses of 1, 2, 3 and 4 Gy using the X-ray generator TITAN-320 (Shimazu, Kyoto, Japan) at a dose rate of ~1.8 Gy/min. After irradiation, cells were plated in 60-mm plastic dishes at the appropriate dilution and cultured for 8 days in medium (DMEM and 10% FBS) with 100 ng/ml wild-type FGF1 or 100 ng/ml FGF1-PIGN alone or with 1 μM PD0325901. After 8 days, the cells were stained with 30% methanol containing 1% methylene blue, and colonies consisting of more than 50 cells were counted. Plating efficiency (PE) was calculated using the following formula: number of colonies/number of cells seeded. Survival fraction was calculated using the following formula: post-irradiated PE/unirradiated PE. To evaluate the ISOS-1 cell radiosensitivity after treatment with wild-type FGF1 or FGF1-PIGN alone or in combination with PD0325901, the D_10_, the dose at which the ratio of colony numbers was 10%, was graphically obtained.

### Cell cycle analysis

ISOS-1 cells were cultured in medium (DMEM and 10% FBS) supplemented with 100 ng/ml wild-type FGF1 or 100 ng/ml FGF1-PIGN alone or with 1 μM PD0325901 for 24 h, then washed with PBS. From this, 1 ml of 1 × 10^6^ cells/ml was prepared. For cell fixation, 1 ml of 70% cold ethanol was added, vortexed and fixed overnight at −10°C. After washing the cells with PBS, they were added to RNase A (Takara Bio Inc., Siga, Japan) and incubated for 30–45 min. Then, 1 mg/ml propidium iodide (PI) (Nacalai tesque, Kyoto, Japan) was added to the cells and stained for 30 min in the dark. After staining, the cells were passed through a 40-μm mesh, with the cell cycle measured and analyzed using a flow cytometer, Gallios (Beckman Coulter Inc., California).

### Statistical analysis

One-way analysis of variance (ANOVA) followed by Tukey’s test was performed to compare three or more groups. Asterisks denote statistical significance (^*^  *P* < 0.05; ^**^  *P* < 0.01).

## RESULTS

### Sustained activation of FGF1 signaling inhibits proliferation of ISOS-1 cells

Wild-type FGF1 and mutant FGF1 (FGF1-PIGN), shown in Fig. 1A, were created and their effects on mouse angiosarcoma cells, ISOS-1 cells, analyzed. In a previous report, 100 ng/ml of wild-type FGF1 or FGF1-PIGN was added to ISOS-1 cells, with the suppression of ISOS-1 cell invasion and migration occurring only in the presence of FGF1-PIGN [15]. Therefore, in this study, to clarify the FGF1-PIGN mechanism of action in ISOS-1 cells, 100 ng/ml of wild-type FGF1 or FGF1-PIGN was added to ISOS-1 cells and cultured. To investigate the effect of FGF1-PIGN on FGF1 signaling in ISOS-1 cells, the expression of ERK, which is phosphorylated (activated) downstream of the FGF receptor (FGFR), was examined by western blot analysis (Fig. 1B and C). When wild-type FGF1 was added to ISOS-1 cells, phosphorylated ERK expression increased at 1 h after incubation but decreased to the same level as that of controls at 12 and 24 h after incubation. In contrast, when FGF1-PIGN was added to ISOS-1 cells, phosphorylated ERK expression was significantly upregulated compared with control and wild-type FGF1 even at 24 h after incubation, and the values were comparable from 1 to 24 h after incubation (Fig. 1C). These results clearly demonstrated that in ISOS-1 cells, FGF1-PIGN activates FGF1 signaling for a longer period compared with wild-type FGF1. The effect of FGF1-PIGN on the proliferation of ISOS-1 cells was examined, and the number of cells was found significantly reduced at 12 and 24 h after incubation with FGF1-PIGN compared with controls (Fig. 1D and E). The relationship between the FGF1-PIGN inhibitory effect on ISOS-1 cell proliferation and FGF1-PIGN concentration was then examined (Supplementary Fig. 1). This showed that the addition of FGF1-PIGN at concentrations of 50 ng/ml or higher significantly inhibited ISOS-1 cell proliferation. Furthermore, the antiproliferative effect of FGF1-PIGN did not increase in a dose-dependent manner when added to ISOS-1 cells at concentrations above 100 ng/ml. In contrast, proliferation inhibition did not occur when wild-type FGF1 was added to ISOS-1 cells (Fig. 1D and E, and Supplementary Fig. 1). The cell cycle was examined and indicated that FGF1-PIGN addition significantly increased the number of ISOS-1 cells in the G2/M phase (Supplementary Fig. 2). In contrast, the cell cycle of ISOS-1 cells was unaffected by the addition of wild-type FGF1. These results indicated that FGF1-PIGN inhibits ISOS-1 cell proliferation by sustained activation of FGF1 signaling.

### Sustained activation of FGF1 signaling strongly inhibits actin polymerization in ISOS-1 cells

**Fig. 2:**
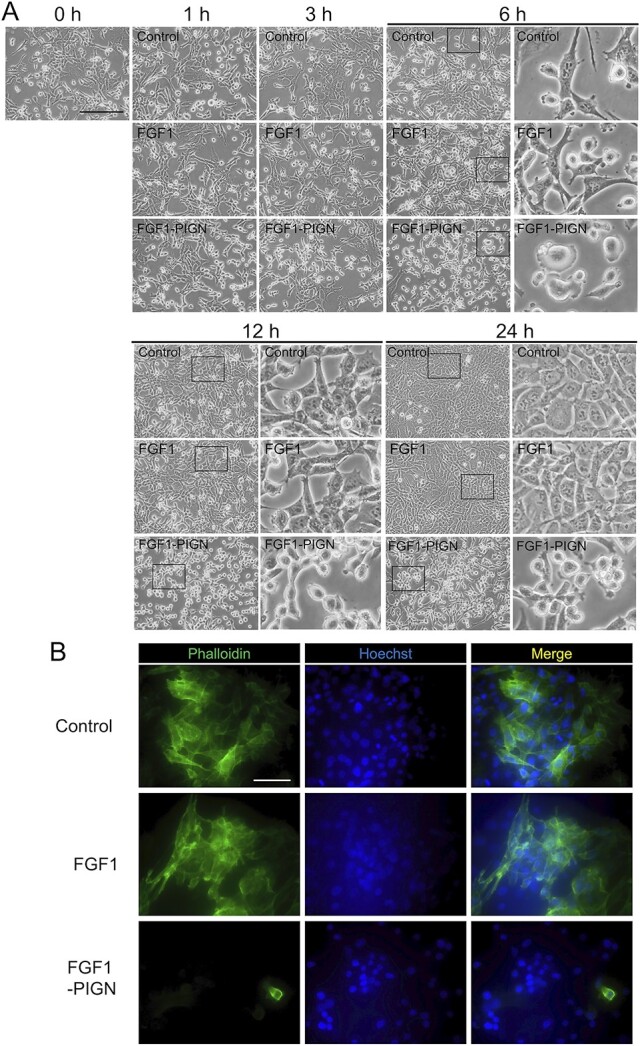
In ISOS-1 cells, FGF1-PIGN potently inhibits actin polymerization. (A) The morphology of ISOS-1 cells cultured for 0, 1, 3, 6, 12, and 24 h with 100 ng/ml wild-type FGF1 or 100 ng/ml FGF1-PIGN. Scale bars: 200 μm. (B) Immunostaining with Alexa Fluor 488-conjugated Phalloidin shows F-actin in ISOS-1 cells cultured for 24 h with 100 ng/ml wild-type FGF1 or 100 ng/ml FGF1-PIGN. Nuclei were stained with Hoechst. Scale bars: 100 μm. Representative images of cell morphology and immunostaining are shown.

Observations of ISOS-1 cells cultured with wild-type FGF1 or FGF1-PIGN showed that culture with FGF1-PIGN induced abnormal cell morphology (Fig. 2A). Normal ISOS-1 cell morphology is spindle-shaped, but 6 h after FGF1-PIGN addition, ISOS-1 cells exhibited a dome-shaped morphology (Fig. 2A). Wild-type FGF1 did not affect ISOS-1 cell morphology compared with the control. Therefore, these results demonstrated that the morphological abnormalities of ISOS-1 cells are an FGF1-PIGN-specific effect.

As ISOS-1 cell morphology was abnormal in the presence of FGF1-PIGN, actin polymerization was investigated, due to the close relationship between morphology and cytoskeleton. Immunostaining showed that actin polymerization was observed in the control or wild-type FGF1-added ISOS-1 cells but was significantly reduced in FGF1-PIGN-added ISOS-1 cells (Fig. 2B). These results indicated that FGF1-PIGN inhibits actin polymerization in ISOS-1 cells through sustained FGF1 signaling activation.

### The inhibitory effect of FGF1-PIGN on ISOS-1 cell proliferation is due to sustained MEK–ERK pathway activation

**Fig. 3:**
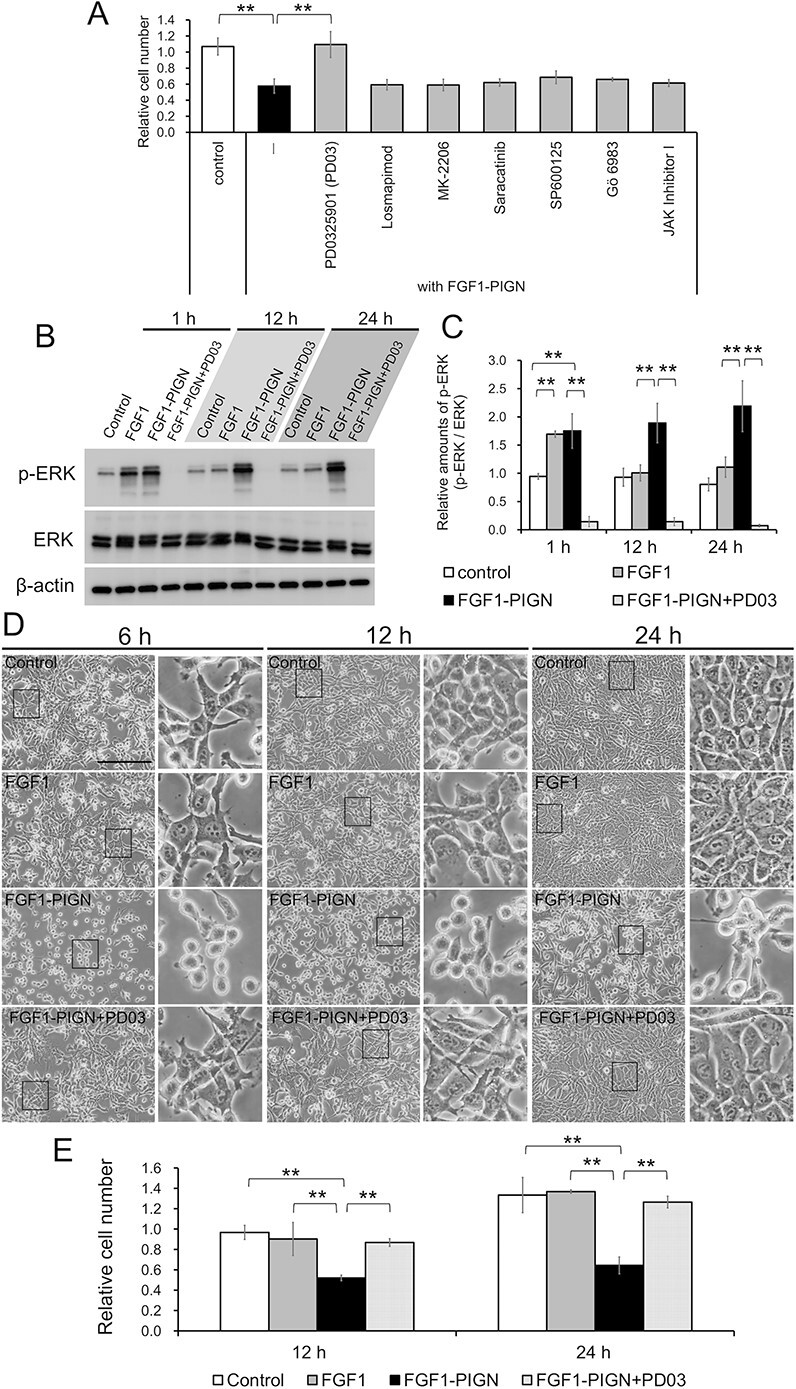
FGF1-PIGN suppresses ISOS-1 cell proliferation through sustained activation of the MEK–ERK signaling pathway. (A) Histograms show the mean number of cells ± SD after normalization against control values (set to 1). Cell counts were performed using ISOS-1 cells cultured with 100 ng/ml FGF1-PIGN alone or with 1 μM PD0325901 (PD03), 1 μM Gö 6983, 5 μM MK-2206, 1 μM Losmapimod, 10 μM SP600125, 1 μM Saracatinib or 0.5 μM JAK Inhibitor I for 24 h. (B) Western blot analysis using antibodies against p-ERK, ERK and β-actin in ISOS-1 cells cultured for 1, 12 and 24 h with 100 ng/ml wild-type FGF1 or 100 ng/ml FGF1-PIGN alone or with 1 μM PD03. (C) Histograms show the mean densitometric reading ±SD of p-ERK/ERK after normalization against levels in control cells after cultivation for 1 h (set to 1). (D) The morphology of ISOS-1 cells cultured for 6, 12 and 24 h with 100 ng/ml wild-type FGF1 or 100 ng/ml FGF1-PIGN alone or with 1 μM PD03. Scale bars: 200 μm. (E) Histograms show the mean number of cells ± SD after normalization against the value of control cells at 12 h (set to 1). Representative images of western blot and cell morphology are shown. Values were obtained from three independent experiments. ^**^P < 0.01.

When FGF1 binds to FGFRs, it activates various intracellular downstream pathways, such as the MEK–ERK, p38 MAPK, phosphatidylinositol-3 kinase-AKT, SRC, JNK, PKC and JAK-signal transducer and activator of transcription pathways [18]. To identify which downstream pathway activation is responsible for ISOS-1 cell growth inhibition by FGF1-PIGN, inhibitors of each downstream pathway were added together with FGF1-PIGN to ISOS-1 cells and cultured (Fig. 3A). Only the addition of PD0325901, a MEK inhibitor, was found to counteract the FGF1-PIGN-induced inhibitory effect on ISOS-1 cell proliferation (Fig. 3A). In addition, western blot analysis revealed that PD0325901 potently inhibited sustained phosphorylation of ERK by FGF1-PIGN in ISOS-1 cells (Fig. 3B and C). Then, whether PD0325901 suppresses cell morphology abnormalities and proliferation inhibition induced by the addition of FGF1-PIGN to ISOS-1 cells was investigated (Fig. 3D and E). ISOS-1 cells cultured with FGF1-PIGN became dome-shaped, while ISOS-1 cells cultured with FGF1-PIGN and PD0325901 remained in a spindle-shaped morphology (Fig. 3D). Additionally, PD0325901 in combination with FGF1-PIGN eliminated the proliferation inhibitory effect of FGF1-PIGN on ISOS-1 cells (Fig. 3E). The addition of FGF1-PIGN significantly increased the number of ISOS-1 cells in G2/M phase, but the cell cycle of ISOS-1 cells was unaffected by the addition of FGF1-PIGN and PD0325901 (Supplementary Fig. 2). These results demonstrated that, in ISOS-1 cells, sustained MEK–ERK signaling pathway activation by FGF1-PIGN caused abnormal cell morphology and proliferation inhibition.

### Sustained activation of the MEK–ERK signaling pathway by FGF1-PIGN inhibits actin polymerization, invasion and migration in ISOS-1 cells

**Fig. 4:**
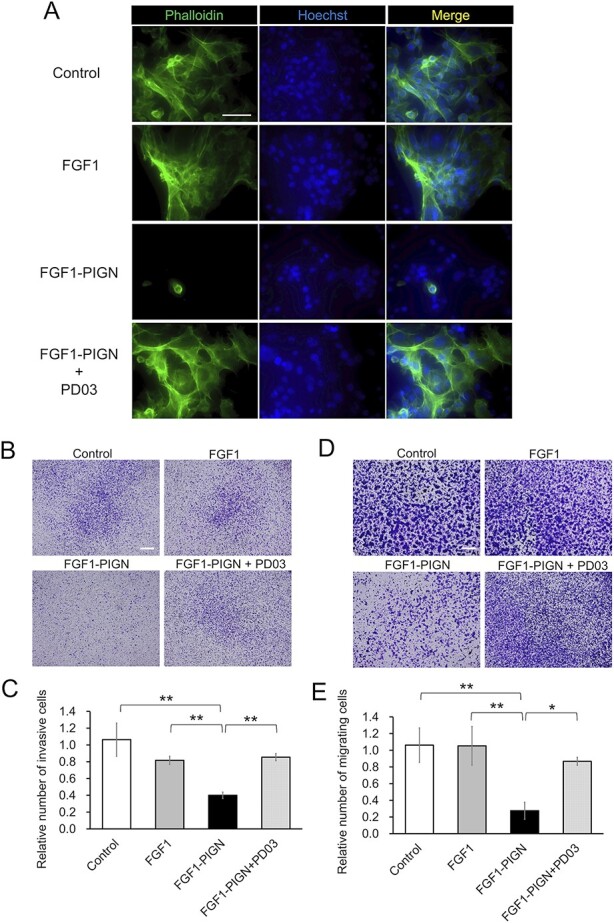
Sustained activation of the MEK–ERK signaling pathway by FGF1-PIGN inhibits actin polymerization, invasion and migration in ISOS-1 cells. (A) Immunostaining with Alexa Fluor 488-conjugated Phalloidin shows F-actin in ISOS-1 cells cultured for 24 h with 100 ng/ml wild-type FGF1 or 100 ng/ml FGF1-PIGN alone or with 1 μM PD03. Nuclei were stained with Hoechst. Scale bars: 100 μm. (B and C) The invasiveness of ISOS-1 cells was examined by invasion assay 24 h after the incubation with 100 ng/ml wild-type FGF1 or 100 ng/ml FGF1-PIGN alone or with 1 μM PD03. Invasive cells on Transwell membrane are shown. The histograms show the mean number of invasive cells ± SD after normalization against the control value (set to 1). Scale bars: 200 μm. (D and E) The migration assay using ISOS-1 cells cultured for 24 h with 100 ng/ml wild-type FGF1 or 100 ng/ml FGF1-PIGN alone or with 1 μM PD03. Migrating cells on Transwell membrane are shown. The histograms show the mean number of migrating cells ± SD after normalization against the control value (set to 1). Scale bars: 200 μm. Representative images of immunostaining and Transwell membrane are shown. Values were obtained from three independent experiments. ^*^P < 0.05, and ^**^P < 0.01.

The suppression by PD0325901 of FGF1-PIGN-induced inhibition of actin polymerization in ISOS-1 cells was investigated. Immunostaining showed that actin polymerization inhibition by FGF1-PIGN was suppressed when FGF1-PIGN was added to ISOS-1 cells in combination with PD0325901 (Fig. 4A). The effects of FGF1-PIGN on ISOS-1 cell invasion and migration capacity were then investigated (Fig. 4B–E). The invasion and migration capacity of ISOS-1 was unaffected by wild-type FGF1, whereas FGF1-PIGN significantly reduced both invasion and migration capacity, results consistent with previous reports (Fig. 4B–E) [15]. Furthermore, the invasion and migration assays showed that PD0325901 counteracted the inhibitory effect of FGF1-PIGN on ISOS-1 cell invasion and migration. These results indicated that in ISOS-1 cells, FGF1-PIGN induces a sustained activation of the MEK–ERK signaling pathway, thus inhibiting actin polymerization, proliferation, invasion and migration.

### Sustained activation of the MEK–ERK signaling pathway by FGF1-PIGN enhances the radiosensitivity of ISOS-1 cells

**Fig. 5:**
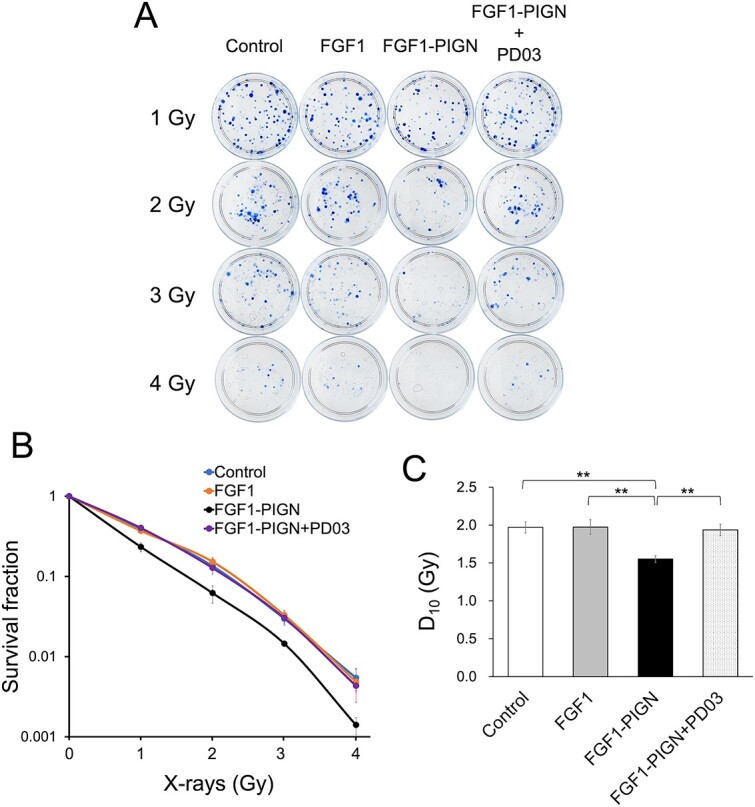
Sustained activation of the MEK–ERK signaling pathway by FGF1-PIGN enhances the radiosensitivity of ISOS-1 cells. (A) Colony formation assay using ISOS-1 cells cultured for 8 days with 100 ng/ml wild-type FGF1 or 100 ng/ml FGF1-PIGN alone or with 1 μM PD03 after irradiation with 0, 1, 2, 3 and 4 Gy. Representative images of colony formation assay are shown. (B) Survival fraction for ISOS-1 cells irradiated with X-rays were assessed by colony formation assay 8 days after the culture with 100 ng/ml wild-type FGF1 or 100 ng/ml FGF1-PIGN alone or with 1 μM PD03. (C) D_10_ is the radiation dose required to reduce the number of colonies to 10%. The histograms show the mean D_10_ value ± SD. Values were obtained from six independent experiments. ^**^P < 0.01.

FGF1-PIGN is previously reported to increase ISOS-1 cell radiosensitivity [15]. Therefore, whether PD0325901 inhibited the increased radiosensitivity of ISOS-1 cells after FGF1-PIGN treatment was examined by colony formation assay (Fig. 5A). The survival fraction of ISOS-1 cells by the colony formation assay after X-irradiation significantly shifted downward by FGF1-PIGN treatment, but were not significantly different when treated with the combination of PD0325901 and FGF1-PIGN compared to controls (Fig. 5B). Furthermore, the FGF1-PIGN treatment reduced the D_10_ value (Fig. 5C). This value indicates the required radiation dose to reduce colony numbers to 10%, while this reduction was not observed for the combined treatment of PD0325901 and FGF1-PIGN. Also, wild-type FGF1 treatment did not increase ISOS-1 cell radiosensitivity (Fig. 5B and C). These results demonstrated that FGF1-PIGN enhances ISOS-1 cell radiosensitivity through sustained MEK–ERK signaling pathway activation.

## DISCUSSION

This study demonstrated that FGF1-PIGN induces a sustained activation of the MEK–ERK pathway in ISOS-1 cells. When FGF1 binds to the FGFR, FGFR is activated by formation of the FGF1-FGFR complex, followed by activation of downstream pathways, such as the MEK–ERK pathway. The FGFR family consists of four members (FGFR1-4) [18], and previous studies showed that FGFR1 is highly expressed in ISOS-1 cells with FGF1-PIGN inhibiting ISOS-1 cell invasion and migration by FGFR1 activation [15]. In addition to previous reports, the present results suggest that FGF1-PIGN suppresses proliferation, invasion and migration and enhances ISOS-1 cell radiosensitivity through sustained activation of the FGFR1-MEK–ERK signaling pathway. Additionally, these results suggest that FGF1-PIGN may be effective in the treatment of cancers expressing high levels of FGFR1. As FGFR1 is highly expressed in human angiosarcoma cells [15], FGF1-PIGN treatment may inhibit the growth, invasion and migration of human angiosarcoma cells as well as ISOS-1 cells. Future analysis of the effects of FGF1-PIGN treatment on human angiosarcoma cells is required.

This study supports the potential of FGF1-PIGN administration as an effective treatment for angiosarcoma. However, as FGF1 administration is generally known to promote cancer cell proliferation, survival and tumor angiogenesis [19], the types of cancer cells inhibited from proliferation, invasion and migration by FGF1-PIGN administration may be limited. It is reported that excessive FGF signaling activation via FGFR1b suppresses the proliferative and metastatic capability of the PANC-1 human pancreatic cancer cell line [20]. As the effect of FGF1-PIGN on cancer cells was only investigated in ISOS-1 cells, further analysis using other cancer cells, such as PANC-1 cells, is necessary. This study demonstrated that the effects of FGF1-PIGN are quite different to those of wild-type FGF1, including cell cycle arrest, actin polymerization inhibition and increased radiosensitivity. Thus, FGF1-PIGN may work inversely to wild-type FGF1 in cancer cells for which FGF1 is reported to promote proliferation and metastasis.

In this study, we showed for the first time the actin polymerization inhibition by sustained MEK–ERK signaling pathway activation in ISOS-1 cells. Several reports exist on actin polymerization inhibition by sustained activation (phosphorylation) of ERK [21, 22]. It has been reported that in mouse and human myogenic cells with mutations in the lamin A/C gene, ERK is constantly phosphorylated with the phosphorylated ERK activating the actin depolymerization factor cofilin-1 by directly phosphorylating the Thr-25 of cofilin-1, thus enhancing actin depolymerization by Thr-25-phosphorylated cofilin-1 [21]. Additionally, phosphorylation of Thr-25 of cofilin-1 by phosphorylated ERK1/2 is also reported to protect cofilin-1 from degradation by the ubiquitination-proteasome pathway [22]. Therefore, in ISOS-1 cells, FGF1-PIGN may promote actin depolymerization through sustained MEK–ERK-cofilin-1 signaling pathway activation. Currently, we are investigating whether cofilin-1 is activated by FGF1-PIGN in ISOS-1 cells. In addition, as the mechanism of actin polymerization inhibition by ERK remains unclear, ISOS-1 cells cultured with FGF1-PIGN may be useful in elucidating the mechanism of ERK-induced inhibition of actin polymerization.

Our findings suggested that cytoskeletal dynamics, such as actin polymerization, are key regulators of proliferation, invasion, migration and radiosensitivity in angiosarcoma cells. Therefore, not only FGF1-PIGN, but also drugs that regulate cytoskeletal dynamics, such as actin polymerization inhibitors, may inhibit angiosarcoma cell proliferation, invasion and migration and enhance radiosensitivity. Actin polymerization is closely related to cancer cell growth and invasion, with actin polymerization inhibitors, such as Latrunculin A and Cytochalasins, reported to inhibit growth, invasion and metastasis of several cancer cells [23–25]. Analysis using mouse pancreatic ductal adenocarcinoma (PDAC) cells showed that the expression of proteins involved in actin polymerization is higher in radioresistant PDAC cells than in radiosensitive PDAC cells, suggesting that actin polymerization inhibitors may be used as radiosensitizers [26]. In human non-small lung cancer cells, Latrunculin A, an actin polymerization inhibitor, is reported to promote radiosensitivity [27]. Additionally, this study also suggests that actin polymerization inhibitors are potential novel anticancer agents, both promoting radiosensitivity and inhibiting cancer cell proliferation, invasion and migration.

In angiosarcoma treatments, the chemoradiotherapy group, which was combined with radiotherapy and taxane anticancer agents, such as Paclitaxel and Docetaxel, had a better prognosis than the combined surgical resection and radiotherapy group [28–31]. Taxanes bind to microtubules that constitute the cytoskeleton, preventing their degradation, thereby inhibiting microtubule dynamics, suppressing cell division and exerting an antitumor effect [32, 33]. In addition, Paclitaxel and Docetaxel not only exhibit cytotoxic effects by inhibiting microtubule polymerization, but also enhance radiosensitivity in various cancer cells by arresting the cell cycle in the most radiosensitive G2/M phase [34, 35]. Actin, as well as microtubules, plays an important role in cytokinesis and entry into mitosis [36]. The actin polymerization inhibitor cytochalasin D has the same effect of arresting the cell cycle in the G2/M phase as Paclitaxel and Docetaxel [37]. In this study, the relationship between the inhibition of actin polymerization by FGF1-PIGN and the enhancement of radiosensitivity by FGF1-PIGN in ISOS-1 cells was unclear. We suggested that sustained activation of the MEK–ERK pathway by FGF1-PIGN in ISOS-1 cells results in G2/M phase arrest (Supplementary Fig. 2). In ISOS-1 cells, FGF1-PIGN strongly inhibits actin polymerization through sustained MEK–ERK pathway activation and may be the mechanism leading to the observed G2/M phase arrest and increased radiosensitivity. The mechanism of action of the radiosensitivity-enhancing effect of FGF1-PIGN on ISOS-1 cells requires further analysis.

To clarify the FGF1-PIGN mechanism of action in ISOS-1 cells, this study used ISOS-1 cells cultured with 100 ng/ml of FGF1-PIGN. Previous studies reported that intraperitoneal administration of 10 μg of FGF1-PIGN to mice 24 h after total-body irradiation with 10 Gy of γ-rays promoted recovery from radiation-induced intestinal injury [15]. In comparison, the FGF1-PIGN dose used in this study is less than that used to treat radiation-induced intestinal injury. As this study examined the effect of FGF1-PIGN on ISOS-1 cells *in vitro*, future *in vivo* analysis using angiosarcoma model mice is required to determine effective FGF1-PIGN dosages for the treatment of angiosarcoma. Several clinical trials used FGF1 doses of 0.03 mg/kg or less [38, 39], which may be useful in determining appropriate FGF1-PIGN dosage with angiosarcoma model mice. The concentration of FGF1-PIGN used in this study is lower than that of FGF1 used in previous clinical trials and animal studies, but safe FGF1-PIGN dosage requires careful consideration as FGF1-PIGN is much more thermally stable than wild-type FGF1, thus will activate downstream pathways for a longer period (Figs 1B and 3B).

In conclusion, we identified a new function of FGF1 signaling in ISOS-1 and demonstrated that sustained MEK–ERK signaling pathway activation by FGF1-PIGN inhibits proliferation, invasion and migration in ISOS-1 cells. We also found that sustained activation of the MEK–ERK signaling pathway by FGF1-PIGN enhances ISOS-1 cell radiosensitivity. Furthermore, we suggested that actin polymerization is closely related to proliferation, invasion, migration and radiosensitivity in ISOS-1 cells. As previous results show that FGF1-PIGN protects the intestine from radiation damage [15], FGF1-PIGN may be a novel anti-angiosarcoma agent that inhibits growth, invasion and migration and enhances radiosensitivity while protecting normal tissue.

## CONFLICT OF INTEREST

The authors declare no conflict of interest.

## FUNDING

This work was supported by JSPS KAKENHI (grant number JP18K15571).

## PRESENTATION AT A CONFERENCE

No work described in this manuscript has been published elsewhere, nor been presented at any conference.

## Supplementary Material

Supplementary_Figure_rrae021
