# Supplementary material for: Sustained activation of the FGF1–MEK–ERK pathway inhibits proliferation, invasion and migration and enhances radiosensitivity in mouse angiosarcoma cells
Source: J Radiat Res. 2024 Apr 18;65(3):303–14. doi: 10.1093/jrr/rrae021 (PMC11115473; doi:10.1093/jrr/rrae021)
Supplement: Supplementary_Figure_rrae021 [file supplementary_figure_rrae021.pdf]

Supplementary Figure

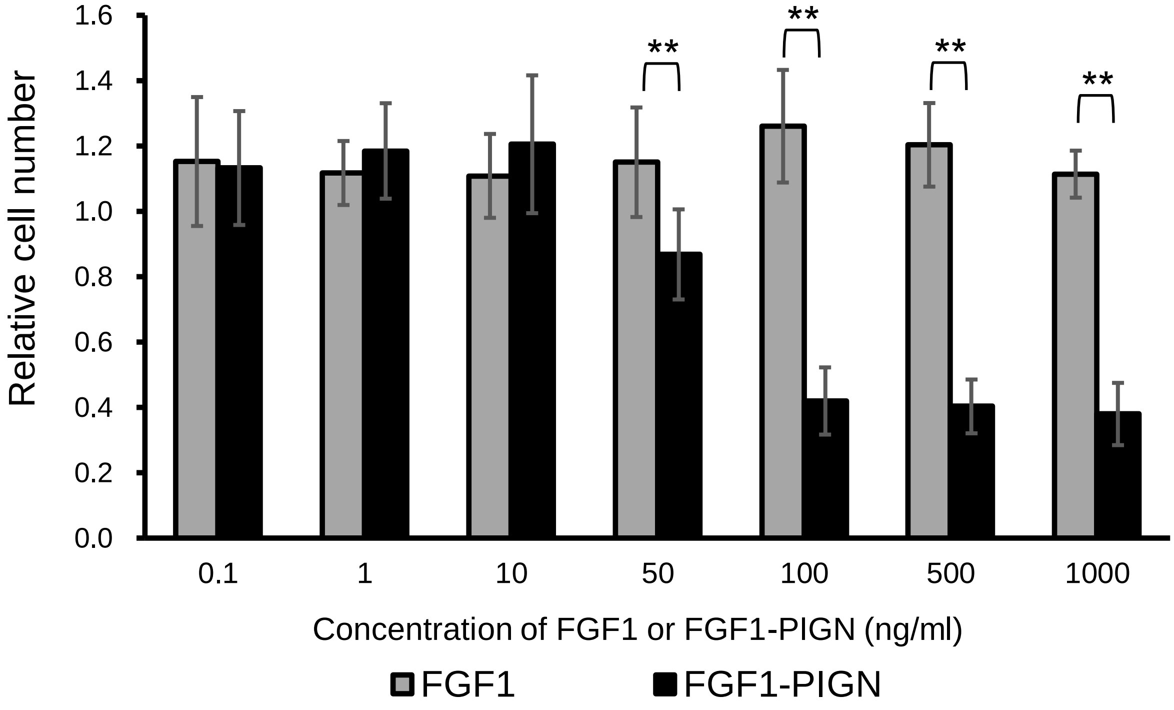

Supplementary Fig. 1. Relationship between the inhibitory effect of FGF1-PIGN on ISOS-1 cell proliferation and the concentration of FGF1-PIGN added to ISOS-1 cells. ISOS-1 cell counts were performed after 24-h culture with 0.1, 1, 10, 50, 100, 500, and 1000 ng/ml of wild-type FGF1 or FGF1-PIGN. Histograms show mean number of cells ± SD after normalization against the number of control cells without addition of wild-type FGF1 or FGF1-PIGN (set to 1). Values were obtained from three independent experiments. ** $P < 0.01$.

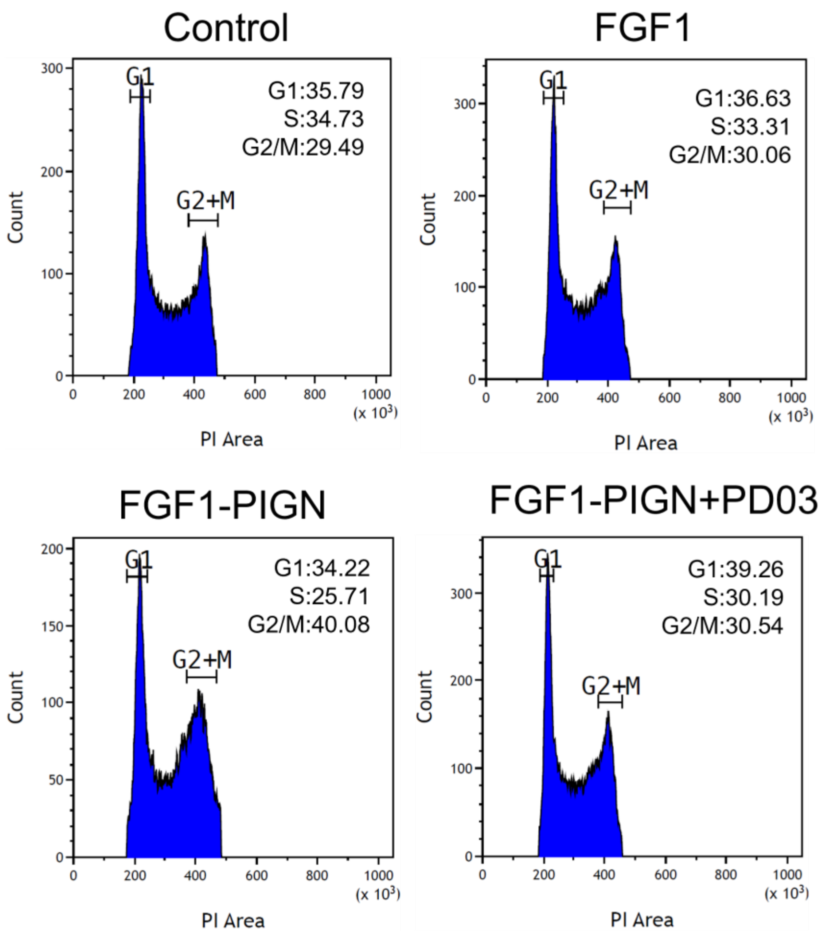

Supplementary Fig. 2. Sustained activation of the MEK-ERK signaling pathway by FGF1-PIGN increases the number of ISOS-1 cells in G2/M phase. ISOS-1 cells were treated with 100 ng/ml wild-type FGF1 or 100 ng/ml FGF1-PIGN alone or with 1 µM PD0325901 (PD03) for 24 h, with cell cycle distribution analyzed by flow cytometer after PI-staining. Representative images of cell cycle analysis are shown. The values shown are the means of three independent experiments.
